# Understanding Urban Bangladeshi Women's Perspectives on Menstruation and Menstrual Products: A Cross-Sectional Survey

**DOI:** 10.7759/cureus.93152

**Published:** 2025-09-24

**Authors:** Farhana Rizwan, Farjana Khatun, Mamunur Rahman, Halima Tus Sadia, Forhad Monjur

**Affiliations:** 1 Department of Pharmacy, East West University, Dhaka, BGD; 2 Department of Hematology and Clinical Pathology, Kidney Foundation Hospital and Research Institute, Dhaka, BGD

**Keywords:** experience, hygiene practice, menstrual product, menstruation, urban bangladeshi women

## Abstract

Background: Menstrual health is vital for women and girls, but it is often overlooked in low- and middle-income countries like Bangladesh. This study aimed to assess the knowledge, attitudes, and practices regarding menstruation and menstrual products among urban women in Bangladesh.

Methods: A cross-sectional study was designed to assess the experience and opinions of women aged 17-45 years. Data were collected via Google Forms (Google LLC, Mountain View, CA) and in-person interviews using a semistructured questionnaire. Descriptive and inferential (chi-square) analyses were performed using Stata version 16 (StataCorp LLC, College Station, TX).

Results: Among 337 participants, 75.4% were aged 20-30 years, and 65.9% were students. Most participants (82.2%) had at least a bachelor’s education, and 74.5% had a monthly income <20,000 Bangladeshi Taka. There was a strong association between income and choice of menstrual product (p < 0.00001). A significantly higher proportion of participants who had received menstrual counseling reported rash and/or itching during menstruation (p = 0.019). Male participants scored significantly lower on menstrual knowledge (p = 0.005), highlighting the need for inclusive awareness and education.

Conclusion: The study emphasizes the need for targeted educational initiatives and policies to enhance menstrual health literacy and ensure access to menstrual products in Bangladesh.

## Introduction

Menstrual hygiene practices are crucial for the health and well-being of women and adolescent girls around the world. On average, 300 million women menstruate each day globally, and they need affordable menstrual health products along with access to water, sanitation, and hygiene facilities to manage their menstruation effectively [1]. Unfortunately, a major portion of these women lack proper knowledge and access to menstrual products and facilities to manage their menstruation in a healthy manner [2]. Menstrual hygiene practices are particularly challenging in low- and middle-income countries (LMICs) as well as for the homeless population throughout the world [3]. In LMICs, cultural taboos and societal stigma often make it difficult to have open discussions about it. This lack of dialogue can lead to insufficient understanding and poor hygiene practices, which can have serious consequences for the physical and psychological health of adolescent women [4]. Research indicates that many young women in Bangladesh face challenges related to menstrual hygiene, due to limited access to sanitary products, inadequate facilities for managing menstruation, and insufficient education on the topic [5].

The significance of menstrual product knowledge should be emphasized, since it has a direct impact on the practices used by women during menstruation. Research reveals that acquaintance with various menstrual products increases understanding and attitudes regarding menstruation, indicating the necessity for hands-on instruction [6]. Furthermore, programs aiming at increasing menstrual hygiene education have produced good results, such as greater knowledge and better management behaviors among school-going girls [7,8]. Despite these efforts, many adolescent women continue to lack adequate understanding. Reports also reveal that a considerable majority of them use unsanitary materials or do not use any protection throughout their menstrual periods [9,10].

In Bangladesh, sociocultural taboos create the perception of menstruation as a shameful phenomenon; only 30%-36% of Bangladeshi women and adolescent girls knew about menstruation before their first period [11]. Moreover, a recent study conducted by Wateraid, Bangladesh, revealed that 94% Bangladeshi women and girls have weak menstrual knowledge [12]. One qualitative study found that even educated urban youth face notable challenges; many participants reported feeling fearful due to inadequate information from their families, and while sanitary pads were the preferred product, embarrassment in purchasing them led some to resort to using old cloths [13]. The sources of information regarding menstruation are often limited to family members, particularly mothers and sisters. Unfortunately, these sources may perpetuate misconceptions due to their lack of knowledge or cultural beliefs surrounding menstruation. This generational gap in understanding can lead to a cycle of misinformation, further exacerbating the challenges young girls face. Additionally, the educational curriculum in Bangladesh often neglects to address menstrual health, leaving many girls without the necessary information to manage their menstruation effectively [8,13]. Though menstrual hygiene is being recognized in urban subpopulations, it exists primarily in specific groups such as among nursing students, not across the spectrum [14].

This study has addressed the existing limited research on menstrual health among urban woman in Bangladesh. The objective of this research was to assess the awareness and practice of urban Bangladeshi women regarding menstruation and menstrual products. To the best of the author’s knowledge, no such prior study specifically conducted only on urban Bangladeshi woman about opinions and experience on menstruation. The objectives of this study are to assess the knowledge, attitudes, and practices regarding menstruation and menstrual products among urban women in Bangladesh and to identify related barriers and socio-cultural influences.

## Materials and methods

Study design

A cross-sectional study was carried out to explore the perspectives of menstrual health and sanitary napkins involving eligible women aged between 17 and 45 years, who lived in urban areas. Women who were pregnant, had gone through menopause, or had a severe illness were not included in this survey. A semistructured questionnaire (English, translated into Bangla) was developed based on previous literature and reviewed by reproductive health experts. It was pilot tested with 20 participants to ensure clarity. Participants were recruited via convenience-based random sampling from various urban areas. Informed consent was obtained from each participant. The study protocol was approved by the Ethical Review Committee of the Department of Pharmacy (ERCDOP) of East West University on January 1, 2023 (approval no. EWU-ERCDOP-A00007). The questionnaire covered sociodemographics, menstrual knowledge, hygiene practices, and product use, and was reviewed by experts in reproductive health to ensure content validity.

Sample size and sample selection criteria

The sample size of this study was determined using the given formula [15]:

$n = \frac{Z^2 \cdot p \cdot (1 - p)}{E^2}$.

Here, Z = 1.96 for a 95% confidence level, and p is the estimated proportion of the population. We assumed a proportion (p) of 0.5 due to the lack of prior data on the prevalence of the key outcome (e.g., adequate menstrual hygiene practices) in this population, as it maximizes the required sample size when an estimated proportion is unknown. E is the error of margin, 0.05 (5.00%). Although a total of 385 samples had been gathered initially according to the equation, only 337 complete responses were used in the final analysis since the rest of the responses contained incomplete or missing information.

Data collection

The study targeted urban women. Convenience-based random sampling was applied in places like markets, offices, and universities in the selected urban locations in Bangladesh. Eligible women were requested to participate if they met the inclusion criteria. The questionnaire was sent via Google Form (Google LLC, Mountain View, CA) on WhatsApp (WhatsApp, Inc., Menlo Park, CA) to English-speaking respondents. Participants with limited English proficiency were interviewed face-to-face by the surveyors who employed a Bangla-translated questionnaire to facilitate understanding. The data were collected from January 2023 to June 2023. Informed consent was taken from each participant at the beginning of information collection.

Data analysis

Data were analyzed using Stata version 16 (StataCorp LLC, College Station, TX). Descriptive statistics (frequencies and percentages for categorical variables; means ± SD for continuous variables) were computed. Inferential analysis was performed using Pearson's chi-square test (or Fisher's exact test when assumptions were not met) to examine associations between categorical variables (e.g., menstrual outcomes and sociodemographic factors). A two-tailed p value of <0.05 was considered statistically significant.

## Results

Among 337 women, 75.4% were between 20 and 30 years of age (Table 1). More than half of the respondents were students (65.9%) and unmarried (65.9%). About three-fourths of the participating women were well-educated, having bachelor's degrees (57.6%) and master's degrees (24.6%). About 59.35% women had less than 10,000 Bangladeshi Taka as their monthly capital for their livelihood, and 84.3% of the women used sanitary napkins or pads during their menstrual period. Details of sociodemographic profiles are represented in Table 1.

**Table 1:** Sociodemographic profiles of the participants BDT: Bangladeshi Taka

Characteristic		Number (percentage)
Age	Below 20 years	35 (10.4%)
	20-30 years	254 (75.4%)
	Above 30 years	48 (14.2%)
Profession	Entrepreneur	12 (3.5%)
	Housewife	36 (10.7%)
	Job holder	67 (19.9%)
	Student	222 (65.9%)
Monthly income/capital	<10,000 BDT	200 (59.4%)
	10,000 to <20,000 BDT	51 (15.1%)
	20,000 to <50,000 BDT	44 (13.1%)
	50,000 to 1,00,000 BDT	26 (7.7%)
	>1,00,000 BDT	16 (4.7%)
Marital status	Married	115 (34.1%)
	Unmarried	222 (65.9%)
Education	No schooling	3 (0.9%)
	Below Secondary School Certificate	18 (5.3%)
	Higher Secondary Certificate	39 (11.6%)
	Bachelor’s degree	194 (57.6%)
	Master’s degree	83 (24.6%)
Trend of menstrual products’ use	Old cloths	26 (7.7%)
	Disposable cotton	23 (6.8%)
	Sanitary pads/napkins	284 (84.3%)
	Tampon	1 (0.3%)
	Menstrual cup	3 (0.9%)

A highly significant association (p < 0.00001) was found between the trend of menstrual products used by the participants and their monthly income/capital to maintain their livelihood (Table 2).

**Table 2:** Association between income and types of menstrual products used by the participants Here, the total number of participants is n = 337. K represents 1,000 BDT in the monthly income ^*^Statistical significance was determined at a p value threshold of <0.05 BDT: Bangladeshi Taka

Menstrual products	Monthly income					p
	<10k (n = 200)	10k to <20k (n = 51)	20k to <50k (n = 44)	50k to 100k (n = 26)	>100k (n = 16)	
Old cloths	24 (12)	0 (0)	2 (4.55)	0 (0)	0 (0)	0.00001^*^
Disposable cotton	19 (9.5)	2 (3.92)	1 (2.27)	1 (3.85)	0 (0)	
Sanitary pad	154 (77)	49 (96.08)	41 (93.18)	25 (96.15)	15 (93.75)	
Menstrual cup	3 (1.5)	0 (0)	0 (0)	0 (0)	0 (0)	
Tampon	0 (0)	0 (0)	0 (0)	0 (0)	1 (6.25)	

Table 3 shows the association between response outcomes of the provided questions and sociodemographic characteristics, such as profession and education. Among all the professionals, statistically significant (p = 0.008) participants obtained counseling regarding menstruation.

**Table 3:** Association between participants' responses and their profession and educational level ^*^Statistical significance was determined at a p value threshold of <0.05 SSC: Secondary School Certificate; HSC: Higher Secondary Certificate; BDT: Bangladeshi Taka

Questions	Response (%)	Profession (n = 337)					Response (%)	Education (N=337)					
		Entrepreneur, n (%)	Housewife, n (%)	Job holder, n (%)	Student, n (%)	p^*^		No schooling, n (%)	SSC, n (%)	HSC, n (%)	Bachelor’s degree, n (%)	Master’s degree, n (%)	p^*^
Q1: Did someone advise you about menstruation?	No (30.3%)	4 (33.3%)	4 (11.1%)	29 (43.3%)	65 (29.3%)	0.008	No (30.26%)	1 (33.3%)	1 (5.6%)	8 (20.5%)	61 (31.4%)	31 (37.4%)	0.059
	Yes (69.7%)	8 (66.7%)	32 (88.9%)	38 (56.7%)	157 (70.7%)		Yes (69.73%)	2 (66.7%)	17 (94.4%)	31 (79.5%)	133 (68.6%)	52 (62.7%)	
Q2: Did you receive any lessons on menstrual hygiene in school or college?	No (75.66%)	11 (91.7%)	28 (77.8%)	54 (80.6%)	162 (73%)	0.319	No (75.66%)	3 (100%)	14 (77.8%)	31 (79.5%)	141 (72.7%)	66 (79.5%)	0.570
	Yes (24.34%)	1 (8.3%)	8 (22.2%)	13 (19.4%)	60 (27%)		Yes (24.33%)	0 (0%)	4 (22.2%)	8 (20.5%)	53 (27.3%)	17 (20.5%)	
Q3: Is it necessary to educate men or boys about menstruation to maintain a healthy environment for a girl or a woman?	No (1.78%)	0 (0%)	1 (2.8%)	1 (1.5%)	4 (1.8%)	0.003	No (1.78%)	0 (0%)	2 (11.1%)	2 (5.1%)	1 (0.5%)	1 (1.2%)	0.005
	Yes (98.21%)	12 (100%)	35 (97.2%)	66 (98.5%)	218 (98.2%)		Yes (98.21%)	3 (100%)	16 (88.9%)	37 (94.9%)	193 (99.5%)	82 (98.8%)	
Q4: Are sanitary pads or napkins too expensive in Bangladesh?	No (14.24%)	3 (25%)	2 (5.6%)	11 (16.4%)	32 (14.4%)	0.305	No (14.24%)	0 (0%)	3 (16.7%)	4 (10.3%)	26 (13.4%)	15 (18.1%)	0.69
	Yes (85.75%)	9 (75%)	34 (94.4%)	56 (83.6%)	190 (85.6%)		Yes (85.75%)	3 (100%)	15 (83.3%)	35 (89.7%)	168 (86.6%)	68 (81.9%)	
Q5: While using sanitary products, have you ever experienced any discomfort or issues?	Itching (20.47%)	1 (8.3%)	4 (11.1%)	20 (29.9%)	44 (19.8%)	0.019	Itching (20.47%)	0 (0%)	1 (5.5%)	3 (7.7%)	42 (21.7%)	23 (27.7%)	0.071
	Rash (64.39%)	8 (66.7%)	31 (86.1%)	34 (50.7%)	144 (64.9%)		Rash (64.39%)	3 (100%)	16 (88.9%)	29 (74.4%)	124 (63.9%)	45 (54.2%)	
	Both (15.13%)	3 (25%)	1 (2.8%)	13 (19.4%)	34 (15.3%)		Both (15.13%)	0 (0%)	1 (5.6%)	7 (17.9%)	28 (14.4%)	15 (18.1%)	
Q6: How much do your sanitary products cost every month?	<200 BDT (62.01%)	5 (41.7%)	27 (75%)	36 (53.7%)	141 (63.5%)	0.194	<200 BDT (62.01%)	2 (66.7%)	15 (83.3%)	23 (58.9%)	129 (66.5%)	40 (48.2%)	0.009
	200-400 BDT (35.32%)	6 (50%)	9 (25%)	28 (41.8%)	76 (34.2%)		200-400 BDT (35.31%)	1 (33.3%)	2 (11.1%)	15 (38.5%)	64 (33%)	37 (44.6%)	
	>400 BDT (2.67%)	1 (8.3%)	0 (0%)	3 (4.5%)	5 (2.3%)		>400 BDT (2.76%)	0 (0%)	1 (5.5%)	1 (2.6%)	1 (0.5%)	6 (7.2%)	
Q7: How often do you change your sanitary pads/napkins in a day?	Every 5 hours (50.44%)	9 (75%)	19 (52.8%)	41 (61.2%)	101 (45.5%)	0.217	Every 5 hours (50.44%)	2 (66.7%)	10 (55.5%)	13 (33.3%)	97 (50%)	48 (57.8%)	0.661
	Every 10 hours (44.95%)	3 (25%)	17 (47.2%)	22 (32.8%)	108 (48.6%)		Every 10 hours (44.51%)	1 (33.3%)	8 (44.4%)	24 (61.5%)	86 (44.3%)	31 (37.3%)	
	Every 13 hours (5.04%)	0 (0%)	0 (0%)	4 (5.9%)	13 (5.9%)		Every 13 hours (5.04%)	0 (0%)	0 (0%)	2 (5.1%)	11 (5.7%)	4 (4.8%)	
Q8: Do you think that your family members are supportive while you are having periods?	No (9.79%)	0 (0%)	4 (11.1%)	6 (8.9%)	23 (10.4%)	0.680	No (9.79%)	1 (33.3%)	3 (16.7%)	5 (12.8%)	18 (9.3%)	6 (7.2%)	0.416
	Yes (90.21%)	12 (100%)	32 (88.9%)	61 (91%)	199 (89.6%)		Yes (90.21%)	2 (66.7%)	15 (83.3%)	34 (87.2%)	176 (90.7%)	77 (92.8%)	

## Discussion

Different educational levels and professions have varying opinions on menstrual hygiene in this study. However, they all agree on the importance of educating men about menstrual health, which is essential for fostering understanding and support in society. Unfortunately, schools and colleges in Bangladesh lack sufficient resources to address this issue.

A news article in Bangladesh claimed that women, particularly those without an independent source of income, are unable to routinely afford sanitary napkins [16]. In Bangladesh, sociocultural taboos and lack of education on menstruation contribute to poor menstrual knowledge; for instance, only about one-third of girls and women knew about menstruation prior to their first period, underscoring the need for better awareness [11]. A recent report highlights that around 86% of rural schoolgirls in Bangladesh lack access to sanitary napkins and resort to unhygienic alternatives [17]. This study revealed that, while the situation has improved, 6.8% continue to rely on disposable cotton, while 7.7% prefer old or reusable clothes. This disparity emphasizes the critical need for access to hygienic menstruation products for all women, regardless of where they live.

In LMICs like Bangladesh, less than half of women and girls use commercially accessible sanitary napkins, compared to over 75% in developed nations, as reported in another study [18]. According to a published report, approximately 71% of Bangladeshi girls and women have used sanitary pads at least once in the previous three menstrual cycles; however, the vast majority still use a combination of cloth and pads, with affordability being the primary factor in their choice [19]. Interestingly, 84.27% of participants in this study reported using sanitary napkins, demonstrating a high need for easily accessible menstrual hygiene products. This study reveals that 69.76% of educated women lack guidance on menstrual hygiene, highlighting the urgent need for better education and support to empower young women and promote their health and well-being.

Sometimes, women do not prefer to purchase it from shops, as most are male-operated [20]. They also use the same sanitary pad for extended periods, which can pose significant health risks, including urinary tract infections [21]. A South Korean study revealed that users reported discomfort and skin rashes associated with sanitary napkins; this finding aligns with the current study, as most participants reported these side effects [22]. This study also unearthed that many respondents routinely use sanitary napkins for long durations and report experiencing itching, rashes, or both during menstruation. Alarmingly, the older generation in Bangladesh often advises younger individuals to use unhygienic cloths due to cultural stigma surrounding menstruation [16]. This outdated mindset can lead to serious infections and health complications. It is crucial to promote awareness about proper menstrual hygiene to ensure the health and well-being of future generations. The Bangladesh government has taken an effective step by implementing a value-added tax exemption on locally produced sanitary napkins, encouraging production and purchasing [16].

Mass education is crucial for fostering awareness about menstruation and its associated hygiene practices. A significant study in Bangladesh demonstrated that structured health education on menstruation increased good menstrual hygiene practices from an alarming 28.8% to an impressive 88.9% [13]. This is not an isolated case; an awareness program in Ethiopia showed that participants not only gained knowledge but also took it upon themselves to educate and empower others, creating a powerful snowball effect [23]. Furthermore, family support during menstruation is vital for the physical and emotional well-being of women and girls in Bangladesh, where cultural norms often discourage open discussions about periods. Such support influences how menstruating individuals perceive and manage their experience, especially in conservative environments. The current study found that 90.2% of respondents had positive thoughts about family assistance during periods.

Our study highlights the urgent need to shift our focus toward understanding the preferences and perspectives of young adults regarding menstrual health. We uncovered statistically significant correlations between responses on menstrual counseling, the necessity of educating males about menstruation, and the experience of adverse effects, all tied to sociodemographic factors like education level, professional background, and monthly income, advocating for enhanced education and accessible resources for all.

Strengths

We note the use of a large urban sample and mixed data collection (online and face-to-face), addressing an understudied population.

Limitations

We acknowledge the cross-sectional design (no causal inference), reliance on self-reported data (possible recall bias), a smaller number of participants, and limited generalizability beyond urban respondents. There was no focus group discussion in this study.

## Conclusions

Menstruation and the use of sanitary napkins are fundamental aspects of women's health. Early detection of abnormalities and understanding of the cycle are crucial to forge a health conscious society. To create significant positive changes in these areas, we must focus on accessibility, education, and affordability. Government organizations and nonprofit organizations should collaborate to conduct campaigns, eliminate stigma, and provide subsidies for these products where applicable. By changing perceptions, addressing stigma spreading awareness, women should be well resourced to manage their menstrual health.
